# Matching-adjusted indirect comparison of guselkumab versus risankizumab in patients with moderate-to-severe plaque psoriasis: Change in baseline Psoriasis Area and Severity Index from week 4 to 40

**DOI:** 10.1016/j.jdin.2023.12.012

**Published:** 2024-01-19

**Authors:** Richard G. Langley, Chiranjeev Sanyal, Aaron Situ, Sarah Alulis, Fareen Hassan, Steve Peterson, Rachel E. Teneralli, Jennifer Lee, Barkha P. Patel, Tim Disher

**Affiliations:** a Division of Clinical Dermatology & Cutaneous Science, Department of Medicine, Dalhousie University, Halifax, Nova Scotia, Canada; b Department of Community Health and Epidemiology, College of Pharmacy, Dalhousie University, Halifax, Nova Scotia, Canada; c CRG-EVERSANA, Value and Evidence Services, Burlington, Ontario, Canada; d Tigermed BDM, Somerset, New Jersey; e Janssen-Cilag A/S, Birkerød, Denmark; f Janssen-Cilag Ltd, High Wycombe, United Kingdom; g Janssen Immunology Global Commercial Strategy Organization, Horsham, Pennsylvania

**Keywords:** change from baseline, guselkumab, indirect treatment comparisons, matching-adjusted indirect comparison, risankizumab, PASI

*To the Editor:*

Multiple biologic treatments are available for patients with psoriasis, an immune-mediated skin disease, including interleukin-23 inhibitors, guselkumab, and risankizumab. Although both demonstrated high efficacy in separate trials, head-to-head studies are lacking. To help inform treatment selection for decision-makers, indirect treatment comparisons can be used to assess comparative clinical effectiveness.

Efficacy in psoriasis trials is evaluated by examining improvement versus baseline Psoriasis Area and Severity Index (PASI) as categorized thresholds of 50%, 75%, 90%, and 100%. However, dichotomizing continuous outcomes (ie, PASI scores), may lead to unreliable conclusions.^1^ Instead, analyzing percent change from baseline (CFB) PASI can provide more precise and clinically appropriate results (Supplementary Fig 1, available via Mendeley at https://data.mendeley.com/datasets/tv5xr9s7xs/1). To compare the efficacy of guselkumab and risankizumab on CFB PASI scores, we used matching-adjusted indirect comparison, a population-adjusted indirect treatment comparisons, which can address cross-trial differences in patient populations by matching individual patient-level data from guselkumab trials to summary-level data reported by comparator trials.^2^

Data from guselkumab (VOYAGE 1 and ECLIPSE) and risankizumab trials (UltIMMa-1 and UltIMMa-2) were selected based on the availability of outcomes and interventions of interest.^3^, ^4^, ^5^ Eligibility criteria and study design were similar across trials, except ECLIPSE was an active comparator-controlled study, whereas the others were placebo-controlled. Because there was no common comparator across the trials, an unanchored matching-adjusted indirect comparison was chosen. Percent CFB in PASI score was analyzed at weeks 4, 8, 12, 16, 28, and 40. Long-term risankizumab data were digitized using DigitizeIt software from supplementary figures with standard errors conservatively imputed through the last observation carried forward when not visible at later time points.

The matching-adjusted indirect comparisons involved matching the guselkumab and risankizumab cohorts on eligibility criteria and reweighting the matched guselkumab patients to align with the characteristics of the risankizumab trials.^2^ Weighting was done using a propensity score algorithm. The relative efficacy of guselkumab to risankizumab was estimated using weighted linear regression models, and the mean difference in percent improvement in PASI score between treatments was calculated.

The primary analysis included all adjustment variables, subsequent analyses removed 1 characteristic at a time to assess their importance, and a sensitivity analysis used only data from UltIMMa-2 to account for lower placebo response in UltIMMa-1. The characteristics requiring adjustment are in Table I; ranking was based on their effect on treatment effect estimates at week 16.^5^

**Table I:** Baseline characteristics—comparison of reported characteristics in matched IPD and comparator trials

Characteristic	Pooled risankizumab∗	Unmatched and unadjusted pooled guselkumab∗	Matched and unadjusted pooled guselkumab∗	Matched and adjusted pooled guselkumab∗
N (sample size, N_eff_)	598	862†	786‡	487§
Age (y), mean (SD)	47.3 (13.5)	45.38 (13.4)	44.93 (13.3)	47.3 (13.5)
BMI (kg/m^2^), mean (SD)	30.5 (7.0)	29.76 (6.8)	29.74 (6.8)	30.5 (7.0)
BSA (%), mean (SD)	26.2 (15.6)	25.49 (14.8)	25.33 (14.7)	26.2 (15.6)
PASI score (0-72), mean (SD)	20.6 (7.7)	20.78 (8.3)	20.77 (8.4)	20.6 (7.7)
PsA, n (%)װ	159 (26.6)	161 (18.7)	142 (18.1)	130 (26.6)
Race— Asian, n (%)	111 (18.6)	69 (8.0)	64 (8.1)	91 (18.6)
Race—Black or African American, n (%)	10 (1.7)	11 (1.3)	9 (1.1)	9 (1.7)
Race—White, n (%)	455 (76.1)	760 (88.2)	691 (87.9)	371 (76.1)
Sex—Female, n (%)¶	183 (30.6)	258 (29.9)	236 (30)	149 (30.6)
IGA or s-PGA: severe, n (%)#	114 (19.1)	203 (23.5)	186 (23.7)	93 (19.1)
Previous biologic use, n (%)	222 (37.1)	227 (26.3)	149 (19.0)	181 (37.1)

*BMI*, Body mass index; *BSA*, body surface area; *IGA*, Investigator’s Global Assessment; *IPD*, individual patient-level data; *LOCF*, last observation carried forward; *N*_*eff*_, effective sample size; *PASI*, Psoriasis Area Severity Index; *PsA*, psoriatic arthritis; *SD*, standard deviation; s-*PGA*, static Physician’s Global Assessment.

∗ Risankizumab (150 mg; Weeks 0, 4, 16, 28, 40) arm of the UltIMMA-1 and 2 trial and guselkumab (100 mg; Weeks 0, 4, then every 8 weeks) arm from the VOYAGE 1 and ECLIPSE trial.

† Values calculated from IPD after removing participants with missing values in the characteristics, LOCF was used to impute patients with missing outcome data.

‡ Matched population includes patients remaining after removing those who would not have been eligible for UltIMMa-1 and 2, based on reported eligibility criteria in Gordon et al (2018).^5^

§ The N_eff_ was reduced to 487 patients after adjusting for all available patient characteristics.

װ Proportion of patients with psoriatic arthritis may not be directly comparable between studies as it is unclear whether studies differed in how diagnoses were adjudicated at enrollment.

¶ Sex was categorized as female or male.

# Proportion of patients with severe disease may not be directly comparable between studies as it is unclear whether studies differed in how diagnosis was confirmed at enrollment.

In primary analyses, the mean percent CFB in PASI was similar for guselkumab in the matched or unadjusted and matched or adjusted cohorts across time points (Fig 1). Guselkumab and risankizumab were associated with comparable mean percent CFB in PASI; treatment differences were small, and their clinical significance unclear. The estimated mean difference of percent CFB in PASI between treatments was 2.89 (95% CI, 0.68-5.10) and 0.32 (95% CI, −1.93 to 2.57) at weeks 16 and 40, respectively (Supplementary Table I available via Mendeley at https://data.mendeley.com/datasets/tv5xr9s7xs/1). Scenario and sensitivity analyses yielded consistent results (Supplementary Figs 2 to 8 available via Mendeley at https://data.mendeley.com/datasets/tv5xr9s7xs/1).

**Fig 1:**
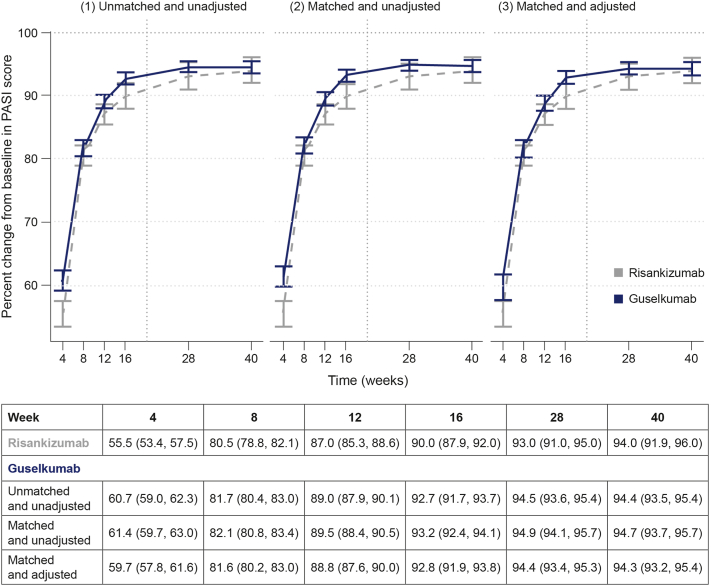
Mean percent change from baseline in PASI score through 40 weeks in patients with psoriasis—primary analysis (UltIMMa-1 + UltIMMa-2).

Previous analyses based on dichotomized response categories may have led to overestimated differences between treatments.^1^ Overall, guselkumab and risankizumab showed comparable efficacy in CFB PASI scores over time. These findings have implications for treatment selection in psoriasis.

## Conflicts of interest

Dr Langley has received honoraria as a principal investigator, scientific advisor, or speaker for AbbVie, Amgen, Boehringer Ingelheim, Bristol-Myers-Squibb, Dermavant, Dermira, Eli Lilly, GlaxoSmithKline, Janssen, Leo Pharma, Novartis, Ortho Dermatologics, Pfizer, Sanofi -Genzyme, Sun Pharma, and UCB Pharma. Drs Sanyal, Disher, and Patel and Author Situ are employees of EVERSANA, which received funding from Janssen Research and Development for this study. Authors Alulis, Hassan, Peterson, and Lee and Dr Teneralli are employees of Janssen Research and Development.
